# The politics of living-with-difference: Local perception of diversity and coexistence around participatory place-making in a multiethnic neighbourhood

**DOI:** 10.1177/23996544231207731

**Published:** 2023-10-06

**Authors:** Byeongsun Ahn

**Affiliations:** 27258 University of Vienna, Austria

**Keywords:** Multilevel governance, participatory planning, social interaction, urban diversity, urban renewal

## Abstract

While much has been said about the structural and spatial dimensions of living-with-difference in the city’s diverse places, existing research has seldom addressed its situatedness within a wider institutional context of place-making that shapes the everyday conditions of our encounters and experiences with ‘others’. As a result, little attention has been paid to the political dynamics of the governance process that engender a context-specific definition and meaning of urban diversity at the local scale. In this light, this article delves into the contextual embeddedness of urban diversity in regenerating a multiethnic neighbourhood, around which residents build their new social relations and belonging. It uses Vienna’s urban renewal model as a research window, through which to explore the political dimension of state-led urban renewal, including institutional and stakeholder arrangements, and its social implications for both old and new residents in everyday spaces. Building on the empirical evidence obtained through field observation and interviewing, it demonstrates how a ‘bottom-linked’ renewal process and its resultant outcome shape a place-specific mode of living-with-difference in the daily life. It concludes highlighting the need for greater attention to the enabling role of the city’s institutional arrangements and policy designs in current research on urban diversity and coexistence.

## Introduction

Emerging from the backlash against multiculturalism in public policy discourse, scholars have criticised the normative description of diversity in governance and public management, and called for a new analytical lens on the multifaceted dimensions of everyday encounters and experiences (Amin, 2002; Gilroy, 2004; Vertovec, 2007). Since this ‘diversity turn’ in the social sciences (see Berg and Sigona, 2013), much has been discussed about the ever-growing demographic complexity of contemporary urban life and its resultant impact on the everyday scene in diverse urban areas. However, existing literature, focusing exclusively on ‘difference’ in shared public spaces (see Neal et al., 2019), has rarely touched upon the political implications of diversity situated within the city’s distinctive institutional arrangements and policy framework, and, thus, its ability to script and regulate everyday experiences and encounters at the local scale. While ethnic plurality and spatial materiality constitute an important element of everyday life in diverse places, there is less information on its contextual embeddedness that forms and reproduces a place-specific narrative of diversity and coexistence – whether positive or negative, according to which people perceive and experience their changing social and physical surroundings.

Indeed, some recent works shed light on the politicisation of diversity in cities and its social repercussions in the daily life. This strand of debate argues that everyday manifestation of diversity embodies the representational framework of the neo-liberal state practices (see Raco, 2018; Ye, 2019). It sees the growing celebration of socio-cultural diversity in urban policy ‘as part of the apparatus of governance and governmentality’ (Ye, 2017: 1042), considering the local perception of diversity as a by-product of the city’s neo-liberal economic restructuring. Such conception of urban diversity, however, mistreats existing institutional and political structures as a mere local expression of global urbanism, undermining deliberate institutional efforts to govern conflicts and problems across historical periods (cf. Sheppard et al., 2013). In fact, there is much evidence that the extent of social cohesion or divide in the context of neighbourhood change may depend on the specific historical and institutional conditions within which government programs unfold (see Silver et al., 2010).

Reflecting on these limitations, this article demonstrates the situatedness of everyday encounters and experiences within the particular institutional context of place-making in which they are configured. Empirically, it draws from the process and outcome of Vienna’s urban renewal policy in a multiethnic neighbourhood, Brunnenviertel, where the participatory place-making process stretching nearly 15 years shaped the specific local experiences with neighbourhood change in its everyday scene. It argues that the local ability to form sociality and forge new social belonging is largely influenced by the city’s specific regulatory capacity, integrating existing institutions, instruments and actors into a congruent policy mix, and providing structural incentives to activate participation of the affected residents. To advance this argument, it shows the facilitative role of ‘bottom-linked governance’ (see Eizaguirre et al., 2012), connecting the respective competences of policy actors and civil society in a new institutional space. As shown below, this complementarity between intervention and participation created a pluralistic local culture, where both old residents and newcomers could preserve their multiple local ownerships in a shared urban infrastructure, and form a new sense of belonging to their changing social environment, without imposing a singular narrative of urban diversity.

The remainder of this article takes the following steps. First, it outlines the three key strands of research on urban diversity and social interaction in everyday life. The aim of this section is to streamline the conflicting – and sometimes overlapping – concerns, arguments and calls for intervention in the current debate. Second, it introduces the case study setting, which details the structural and institutional contexts behind the state-led urban renewal of Brunnenviertel. This is followed by a section on the methodological framework of this article. The fifth and sixth sections present the major empirical findings, which illustrate the integrative effects of instrumental policy-making on the local perception of diversity and coexistence in everyday spaces. The final section discusses these findings in relation to their scholarly and practical implications.

## Researching urban diversity and social interaction in everyday life

In the context of the new demographic reality in globalising cities, the recent writing on urban diversity and coexistence explored the normalcy of ‘super-diversity’ and its translation into ambiguous attitudes towards ‘others’ in everyday life (see Neal et al., 2019; Nowicka and Vertovec, 2014; Wessendorf, 2014). Echoing the earlier literature on everyday urbanism in shared public spaces (see Amin, 2002; Anderson, 2004; Crawford, 1999), everyday practices of living-with-difference are said to characterise the place-based ethics of public civility, whereby individuals remain indifferent to each other in their short-lived encounters (Jones et al., 2015; Radice, 2016; Wise and Velayutham, 2014). At the same time, they reveal the negative dialectics of ‘indifference-to-difference’, which lies in tandem with commonplace intolerance. It is for this reason that this line of debate situates everyday encounters and experiences with ‘others’ at the crossroad between pre-existing conditions of aversion and the framing of migration in mainstream politics (Karner and Parker, 2011; Tyler, 2017; Valluvan, 2016; Wessendorf, 2014).

Concurrent with its widespread use across different fields of social science, however, those, who argue against ‘super-diversity as a methodological lens’, cautioned that the abstraction of urban complexity into migration-driven social diversification obscures existing structural inequalities, political powers or policy concerns (see Aptekar, 2019; Hall, 2017; Sealy, 2018). Such a narrow conception of everyday life underestimates the gap between micro-scale interpersonal connections and entrenched prejudices towards minority groups. In reality, the ethics of ‘indifference-to-difference’ in fleeting encounters might not sustain the same intensity of acceptance to create a meaningful intercultural dialogue between community-based narratives.

In this light, scholars, who underline structural inequalities in everyday spaces, criticised the cosmopolitan bias towards intercultural competence in the ‘super-diversity’ literature (see Valentine, 2008). Considering social prejudices that are deeply rooted in specific histories and geographies, this second key line of debate perceives that the local perception towards diversity is conditioned by other intervening social contexts, such as the lived-experiences of migration (Gawlewicz, 2016); spatial configurations of contact zones (Mayblin et al., 2015); or socialisation experiences with minorities (Piekut and Valentine, 2017). Therefore, it warns of the descriptive naivety in the literature for misinforming policy-making, which underestimates more fundamental social issues in action (see Matejskova and Leitner, 2011). Instead, it calls for a public policy that enhances the ‘perceived fairness of resource distribution between majority and minority populations’ (Valentine, 2008: 334), connecting social prejudice towards minority groups with the dynamics of urban politics (see Rocha and Espino, 2009).

This approach is useful in addressing the spatial and temporal sensitivity of living-with-difference, but tells little about its embeddedness within the intersecting socioeconomic and -political processes that shape the very nature of existing inequalities and the resultant conditions for social interactions (see Raco, 2018; Ye, 2019). While the spatial and temporal settings of diverse places may reveal the extent of intercultural tolerance in micro-scale interactions, they do not explain the political narrative of urban diversity within a wider institutional context of place-making that breeds particular imaginaries of living-with-difference in urban life.

Against these shortcomings, a growing body of literature has reflected on the politicisation of diversity in the context of building creative and competitive cities, forming a simplified – and selective – narrative of multiculturalism in policy-making and, as a result, creating particular social imaginaries about ‘others’ (Hoekstra, 2018; Saeys et al., 2019; Scott and Sohn, 2019). This is the third key line of debate that interprets the local perception of diversity and changing social surroundings as emerging from the prevailing shift towards neo-liberal governance, where certain behaviours and expectations of ‘others’ are imposed by public interventions in different social spaces of everyday life, such as employment (Yeoh, 2006), mobility (Ye, 2017), or residence (Collins, 2016). Arguing that negative outgroup attitudes feed off growing inequalities in the built environment of creative growth and development, it calls for political actions that elevate existing urban conditions, ‘promot(ing) more positive modes of encounter…regulari(s)ed, repeated, and institutionally-mediated social interactions in breaking down barriers to difference’ (Raco, 2018: 158).

Those in this argument share an overall pessimistic view of public policy-making, perceiving the thin celebration of diversity in globalising neo-liberal discourse to exacerbate existing inequalities in the city and, as a result, deepen social divides in everyday life. Yet, their overemphasis on the economic rationality of place-making tends to disregard the distinctive capacity of public institutions to response to emerging urban problems in their own way and, therefore, their ability to mitigate the resultant social effects at the local scale. This conceptual overstretching has another practical issue. While this strand of literature calls upon civil society to mobilise against the dominant governance culture, and state actors to reduce structural inequalities, it remains unclear, as to which recognition and redistribution are at all possible for whom and by whom – and to what extent – within existing local institutional arrangements. As a result, it obscures more practical issues that pertain to the complexity of the multilevel governance process in a real-world situation.

In fact, the city’s diverse places are not neutral containers of encounter, inequality or change. Rather, these are curated spaces of scripted contacts, social relations and complex policy-making, intersecting with various arrangements between institutions and actors, and their relations in the governance process, all of which are particular to the local scale in question. Considering this contextual embeddedness, the city’s regulatory capacity plays an important mediating role for both physical and social outcomes of neighbourhood change, preserving existing places of multiple social identities and mitigating the adverse impact of structural inequalities in their interactions. This bears significance for attenuating social tensions and conflicts between residents, as the ability and availability to sustain their own locations of identity become conducive to positive perception of living-with-difference (see Pemberton and Phillimore, 2018). It is in this wider context of place-making that this article situates the local perception of diversity and coexistence within the city’s institutional arrangements and their fit in the specific policy design, shaping the precise way, in which these are encountered and experienced in everyday spaces.

## Case study setting

Since Vienna’s overall decline in the 1970s, the decaying urban infrastructure and the high number of substandard apartments characterised much of the living condition in the city’s inner-city districts (see Hatz, 2009). Notwithstanding sustained growth in population and increasing investments in housing from the late 1980s onwards, some areas became more vulnerable to the city’s structural shift, especially due to the large influx of low-skilled migrants and their segregation in low-quality housing (Friesenecker and Kazepov, 2021). Furthermore, the combined influence of limited access to subsidised housing and growing free market rental housing resulted in the spatial clustering of low-income migrants in the private rental sector along the fringes of the inner-city districts. These pockets of poverty, featuring under-equipped and ill-maintained affordable housing, reinforced the residential segregation among low-income migrant families without access to subsidised housing nor sufficient financial resources for private housing.

Brunnenviertel serves as a typical example of this historical trend. It is a multiethnic neighbourhood, located in the city’s second most diverse district, Ottakring, with a large number of high-density housing blocks from the late-19^th^ century on the western edge of the inner-city areas. Following the steep decline associated with deindustrialisation and suburbanisation, it remained one of Vienna’s most disadvantaged neighbourhoods well into the 1990s with more than 40% of its dwelling units lacked indoor plumbing and a floor space above 45 m^2^ (see *Building Condition – Category D* in Table 2). Due to the rent regulation based on the equipment standards of housing, this poor living condition kept the average rent price lower than elsewhere in Vienna (see Rode et al., 2010). The availability of low-cost housing contributed to the larger presence of migrant residents – mostly from the former Yugoslavia and Turkey – than the city average (1991: 26.68%, Vienna 12.77%), among whom only a marginal proportion received higher education (see *Educational Attainment* in Table 1).

**Table 1.** Sociodemographic changes in brunnenviertel and its surrounding statistical enumeration districts, 1991–2017.

	Residents	Nationality	Educational attainment	Nationality	Educational attainment
	Total	Austrian %	Tertiary % of Austrians	Foreign %	Tertiary % of Foreigners
1991	8,651	73.32	6.69	26.68	5.43
2001	8,083	66.05	11.52	33.95	6.10
2011	8,730	63.79	24.28	36.21	14.39
2012	8,780	62.94	26.10	37.06	15.12
2013	8,908	62.14	28.19	37.86	15.69
2014	8,938	60.92	30.33	39.08	17.35
2015	9,231	59.38	31.94	40.62	17.21
2016	9,490	57.77	33.75	42.23	17.12
2017	9,750	56.17	34.92	43.83	17.89

Source: Author’s own calculation based on Register-based Census Data.

**Figure 1.**
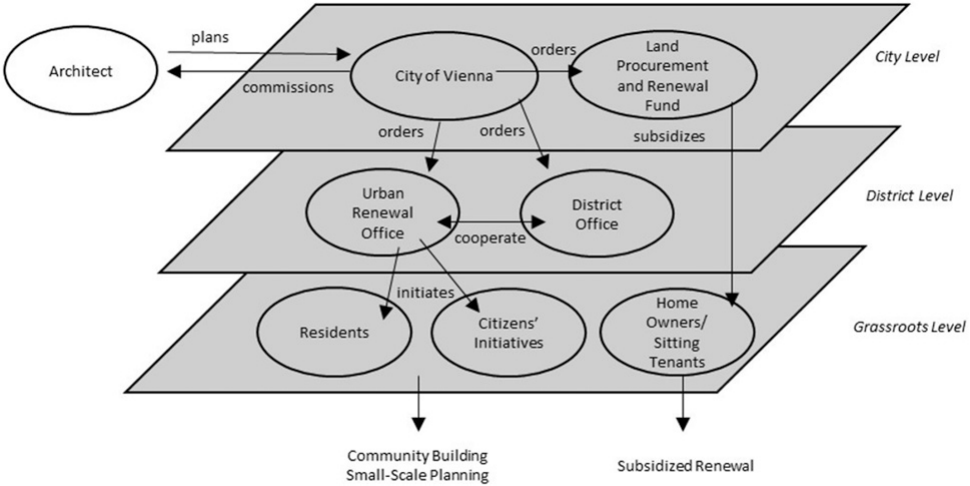
Multilevel participatory urban renewal in vienna. source: Author’s own elaboration.

Brunnenviertel and its surrounding areas underwent a major renewal process between 1997 and 2010. In brief, the city’s urban renewal model, also known as Soft Urban Renewal (*sanfte Stadterneuerung*), occurs typically through two channels of participation, which are organised by two separate public institutions that operate at two different territorial levels (see Figure 1). First, the city’s own housing fund, Vienna Land Procurement and Urban Renewal Fund (*wohnfonds_wien*) provides subsidies to private homeowners for physical improvement of dilapidated housing stocks in renewal areas. The specific renewal mechanism here is the rent control imposed on upgraded apartments after subsidised renovation, which aims at preventing physical displacement of sitting tenants and mitigating land speculation by private developers in the overall neighbourhood. Second, the Urban Renewal Office (*Gebietsbetreuung Stadterneuerung*) coordinates resident participation in neighbourhood planning. While controlled by the city’s own renewal department, it solely operates at the district level in corporation with the district authorities, who have full or partial jurisdiction with their own budget to self-govern small-scale planning activities. This social element comprises the second pillar of Soft Urban Renewal, which aims at preventing social displacement and facilitating community-building between old and new residents in neighbourhood regeneration.

These structural and institutional contexts produced the configuration of the local specificities, from which the particular aims and objectives – and their narrative – of regenerating Brunnenviertel emerged. Yet, these conjoining factors must be brought into an institutional space, where a specific mix of existing policy instruments are put together in a congruent way that exerts an anticipated effect for target groups and their behaviour. As we will see, it is the result of this specific design and its designing in the process around which all residents ultimately form their everyday lives and experiences as the outcome.

## Methods and data collection

The collection of the empirical data presented in this article followed three steps. First, a document analysis of the three policy reports from the City of Vienna captured the key contextual information about the institutional design and instrument choices to achieve the stated aims and objectives of regenerating Brunnenviertel. It identified: a) the structural and political circumstances, from which the specific renewal narrative – and that of urban diversity and coexistence – entered onto the agenda-setting process; b) the design and designing of a new institutional space, in which this narrative is implemented; and c) the specific empirical content and activities of this design in the actual renewal process.

Second, nine expert interviews were held with the key stakeholders – both institutional and grassroots – at different levels of the city’s renewal governance, all of whom were directly involved in the renewal of Brunnenviertel. The sample included three planners from the city’s planning department, two planners from the district’s Urban Renewal Office, and three activists who initiated the first grassroots movements prior to the official renewal process. Additionally, one interview was conducted with the main coordinator of a cultural initiative, which currently organises participatory activities in the neighbourhood. These interviews elicited information about their varying interests, motivations, and goals of promoting urban diversity and citizen participation, revealing the specific institutional logic of the overall renewal process.

Third, field work was conducted in various semi-public and public spaces in Brunnenviertel between January and August 2019. This phase captured the perception among both old and new residents towards the changing social and physical environment in the neighbourhood. Prior to conducting interviews, three semi-public spaces in the neighbourhood were routinely visited, where contacts were established, and informal conversations with the occupants were recorded in fieldnotes. Among those whom I acquainted with through multiple visits to the three sites, ten semi-structured interviews were carried out, eliciting the concrete ‘episodes’ of their lived-experience from interacting with other residents and visitors in the renewed locations. The recruitment of nine other respondents was done through convenience sampling in public spaces. Here, the interviews followed a causal, unstructured fashion with open-ended questions.

## Making of Brunnenviertel: Design and designing of a new institutional space

Since the first inception of Soft Urban Renewal in 1974, Brunnenviertel and its surrounding areas experienced a number of small-scale renewal activities, which, however, had little significance for achieving its social aims of resident participation and community-building (see Hatz, 2021). One particular issue at stake was the fragmentation between the key institutions and actors in the renewal process, reinforced by the lack of an overarching policy framework to coordinate the increasingly diversified and decentralised renewal instruments. Offloading responsibilities to the Urban Renewal Offices and the district authorities ensued without matching financial resources and decision-making capacity in the renewal process, of which concept and strategies were still formulated at the city level. While the renewal concept required a significant level of homeowner participation, encouraged by extensive financial subsidies, that of sitting tenants and other relevant local actors only occurred once it was already in the works (Fröhlich, 1992). In effect, participation of homeowners and residents – and their interaction with the key institutions – happened separately in different phases of the renewal process.

**Figure 2.**
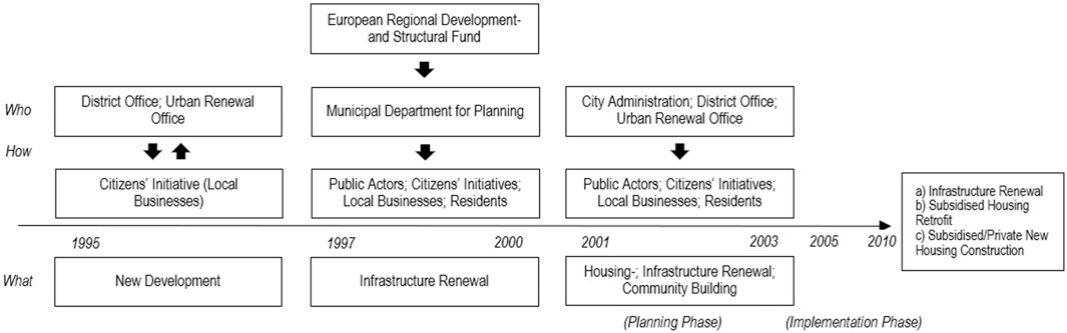
Process of Soft Urban Renewal in Brunnenviertel. Source: Author’s own elaboration based on Municipal Department 18 – Urban Development and Planning (2000); Municipal Department 18 – Urban Development and Planning (2004); Municipal Department 27 – European Affairs (2002) and Rode et al. (2010).

These specific institutional and actor arrangements of Soft Urban Renewal had a great design implication for formulating a new multilevel renewal process in Brunnenviertel. Its first major redevelopment began within the framework of the URBAN Community Initiative by the European Reginal Development in 1997. In line with the funding guidelines requiring active resident participation in urban renewal and community-building, a new organisational structure of participatory renewal was developed for Brunnenviertel’s public square, Yppenplatz, with the three core aims of open space creation, a new street market- and traffic concept (Municipal Department 18 – Urban Development and Planning, 2000). In contrast to the city’s standard renewal process, this new design package, enabled by the co-financing from diverse public institutions and interest groups at both the national and city level, created a new functional management structure, incorporating a diverse range of both public and private stakeholders into working groups around the three renewal aims (see Figure 2). The working groups composed of the city’s municipal departments, the district office, external experts in the public sector, grassroots initiatives, residents and local businesses, whose representatives co-decided the final renewal plans with the commissioned architectural firms.

These plans were subsequently turned into concrete renewal projects based on the three major themes: open space creation, street market regeneration and housing renovation. Such large-scale renewal projects, which required interdepartmental coordination between multiple public institutions with varying policy competencies, were enabled by a diversified financial structure, mobilising resources and capacities from various governance levels. The financial contribution of the district office and the interest groups representing the local businesses gave the local-level stakeholders significant decision-making authority in designing renewal activities that normally exceeded their formal responsibility.

On the one hand, the new policy capacity created at the district level allowed the Urban Renewal Office to take an active role in organising and steering the interaction between the homeowners, the tenants and the city’s housing fund, subsidising more than 70% of the entire housing renovation that took place between 2000 and 2008 (Rode et al., 2010). Hans, an urban planner at the district’s Urban Renewal Office, described their active steering role in regenerating Brunnenviertel as an important tool for mitigating potential conflict between different stakeholders with multiple interests in the agenda-setting process:

(the urban renewal office) functioned as a knot that connected the city and the district, the planners and the residents, and the business owners and the homeowners. Our goal was to create a space for participation of all these different groups, so that people equally share the responsibility. Steering and planning committees were set up, information was distributed, public meetings were held, and all these would feed back into additional rounds of discussion. They were a lot of different interest groups involved, who were interested in different things...But, after a years-long effort, we came to an agreement on a ’10-point’ program for top priorities of Brunnenviertel’s redevelopment.

On the other hand, the diversified funding streams facilitated the participation of grassroots initiatives in the street market regeneration and its surrounding public open spaces, giving structural incentives to a diverse range of cultural and business activities to fill the vacant retail spaces (Schneider, 2008). For Theresa, a local activist behind the main grassroots initiative in the renewal process, the diversity of available resources played an important role for their community-building activities especially when they came into conflict with more profit-driven renewal interests:

As the time passed, we felt like we were treated like a PR tool (of the Economic Chamber). They were interested in bringing in businesses into Brunnenviertel, but not so much the migrant businesses that were already there. So, we had to find another way (to fund) our activities…We were lucky, because not only the city and the district supported us, but also the federal government and the Chamber of Labour. We did a lot with this money for many years.

The top-down supports for the bottom-up driven initiatives enabled the actual implementation of community-building and local economic regeneration, to which substantive participation of the residents and the business owners was vital. Their active engagement created not only a physical platform for self-organised participation, but also a social network between community actors, such as local artist and business associations, promoting actually existing diversity of the neighbourhood as the main driver of its social transformation. Combined with the subsidised physical renovation, this social element of Soft Urban Renewal, creating a new neighbourhood image through grassroots organisations, had a significant effect on the local economic regeneration, as Hans mentions:

(Brunnenviertel) lacked a technical infrastructure and didn’t meet any hygienic standards neither as a market nor as a living space. The residents here were mostly old, poor, and refugees…Back then, Brunnenviertel was considered as the worst place in the city. At least, that was its image…Our main objective was to invite a new audience to the neighbourhood. But, not the audience that was too poor. We wanted better social mix in the neighbourhood…with a multicultural touch for a new image…And the local shop owners are happy that very mixed visitors now come to the market. Brunnenmarkt is now the most profitable market in the entire city.

Between 2005 and 2010, Brunnenviertel underwent additional renewal processes with a specific focus on the expansion of the pedestrian zone and public seating, and the quality management of the market products.

The design context, from which this renewed image emerged, highlights the importance of the city’s regulatory capacity that was able to shape the specific conditions of urban diversity and coexistence in an instrumental way, in which the instruments and mechanisms produced their expected outcomes. It influenced its capability to configure existing renewal institutions across multiple scales in a right temporal moment and mix their policy instruments with strong structural incentives, mitigating the negative spillover effects of neighbourhood regeneration that are associated with physical or social displacement. In fact, the major observable change that took place is the qualitative upgrading of housing units, where the number of substandard- and small apartments halved between 1991 and 2011 (see *Condition* and *Size* in Table 2). That said, no necessary correlation between physical regeneration and social displacement is visible, as shown in the stability of the tenure structure and resident characteristics (see *Occupancy Type* in Table 2 and Nationality in Table 1). This partly also owes to the fact that most of the housing units in Brunnenviertel were built before 1945, which means nearly 70% of them are rent-regulated in one way or another.

**Table 2.** Changes in living and housing conditions in Brunnenviertel and its surrounding statistical enumeration districts, 1981-2011.

	Occupancy type		Condition				Size (m^2^)				
	Ownership	Tenancy	A	B	C	D	<45	45–60	60–90	90–130	130>
	%		%				%				
1981	20.10	71.36	n.a	n.a	n.a	n.a	48.87	19.04	24.60	6.21	1.28
1991	19.70	68.16	44.13	9.89	5.10	40.88	39.74	22.59	28.40	8.07	1.21
2001	20.87	69.34	67.63	9.15	1.80	21.41	33.77	23.13	32.72	9.20	1.19
2011	20.94	70.28	75.55	3.76	1.50	19.19	29.15	27.75	33.31	8.59	1.21

Source: Author’s own calculation based on Register-based Census Data.

Note: A: fully equipped with a floor space of at least 30m^2^; B: partially equipped without a stationary heating system; C: only with indoor water supply and toilet; D: without either indoor water supply or toilet, or both.

The empirical findings below present the place-specific mode of living-with-difference that is organised, encountered and experienced around these particular outcomes of Soft Urban Renewal in Brunnenviertel. They show the social implications of this regulatory capacity in neighbourhood regeneration for the local culture, born out of the specific institutional context of its place-making, which nurtures multiple social identities in a shared urban infrastructure without leaving a particular group excluded from the local language of diversity and coexistence.

## Local perception of diversity around the politics of place-making

Everyday encounters and experiences with ‘others’ in Brunnenviertel’s shared public spaces entail a sense of togetherness that the residents and visitors have formed around its new image of an inclusive ‘multicultural’ neighbourhood. This new image engenders a sense of belonging to a changing neighbourhood, where they appreciate its openness and celebrate its diversity. Otto, an urban planner from the city’s planning department, attributes this to a feeling of pride among the residents, who now perceive their neighbourhood in a different light:

(Before the 1990s) Not only did Brunnenviertel have a bad image, but also a bad identity…After cultural initiatives began…people started to take an interest in the neighbourhood. A specific clientele took an interest, and (artists) began to change its image that, not only they, but also the residents identified themselves with. The residents realised that they no more lived in a bad neighbourhood in a fringe district of the city. The entire city was interested in Brunnenviertel. They realised this interest…They didn’t have to hide that they lived in this neighbourhood anymore, and they could be proud of it.

For the residents, this new image plays a significant role in the way, in which they perceive existing diversity in the neighbourhood and shape their interactions with ‘others’ in its everyday scene. Brunnenviertel is no more a dilapidated ‘problem zone’, but the symbol of lively, urban, and cosmopolitan Vienna, where people from different districts travel to experience the city’s multicultural competence. Karl, an old ‘native’, who has witnessed this transformation from its beginning, commented on this shift:

(The City of Vienna) did a good job. All the shops you see down the market were bad. Really bad. It was always empty, and people never came here before. But now people even travel from different districts to come here... When the weather is nice, I go and sit on (Yppenplatz) to enjoy the sun. I don’t really care, who comes here. Anyone can come. I mean, I don’t own this place, do I?

This feeling of togetherness in a place for ‘everyone’ constitutes an important aspect of everyday life in Brunnenviertel, which provides a diverse range of social groups a sense of belonging to the neighbourhood. For many young visitors and residents, this sense of belonging to Brunnenviertel emerges from the excitement of being part of the city’s most attractive urban area, where they celebrate their physical proximity to diversity. The presence of ‘difference’ in shared public spaces is central to their own sense of belonging to the neighbourhood. On the day of Brunnenviertel’s annual multicultural festival, Lukas, a young ‘native’ said:

Of course, I contributed to ‘gentrification’ in Brunnenviertel…I’m a ‘gentrifier’ in that sense. But do you remember, how it used to be? A couple years ago, no one ever talked about this place. But, now look (at Yppenplatz). This place is so full! There is always something going on here. Migrants, hipsters, kids, old people…They do all different stuff, and with their own people. But they do it all on this square!

[…]

I love this neighbourhood, because this is the most exciting place in the city. I love, how I can just sit down (on Yppenplatz) with friends and have a beer, right after I go shop on the market. I live nearby, but, more importantly, there are bars and shops, where I go regularly with friends. That’s why I come here almost every day.

The extent to which the locals identify themselves with the changing surroundings of Brunnenviertel emerges not only from its demographic composition nor its physical environment itself alone. Rather, it is the local ability and availability to nurture existing social identities from their changing social and physical surroundings, where no particular social group is, however, privileged or disadvantaged, as Otto explained:

Not everything in Brunnenviertel is made completely new. Public spaces were already there, and so was ethnic diversity. Before diversity became a catchword, the residents of Brunnenviertel were always ethnically diverse. And it remains so. Of course, it was then perceived differently…but for (public actors) it was always clear that this was an ethnically diverse neighbourhood…We do not serve a specific social group, nor do we create a specific space for them. Our job is always to create spaces for everyone, while paying attention to minorities who might require a special attention.

For this communal environment, the local grassroots initiatives are instrumental in creating and curating physical spaces of encounters that guides different social groups to come together as a community, who otherwise rarely come into meaningful contacts. While the city’s strong intervention shaped the structural conditions for positive encounters and experiences with neighbourhood change, the active engagement of grassroots actors engendered the mutual responsiveness and -responsibility at the local level. Indeed, this synergy, complementing the knowledge and capacities from both bottom-up and top-down, is vital to ‘overcome the participation trap of loosely coupled participatory processes’ (Van Meerkerk, 2019). This provides an important social context for the community-based renewal process, whereby the newcomers themselves incorporate existing diversity into their new sense of belonging to the neighbourhood. As a long-term ‘native’ resident, Wolfgang, who led the neighbourhoods’ first citizens’ initiative in the mid-1990s, recalled:

Shortly after (I moved here), a development plan surfaced and the district talked about getting rid of the street market for a multi-storey apartment building with a market hall…But (Brunnenviertel) wasn’t going to get better, if only Austrians were to move into the new apartments. A lot of people (in Brunnenviertel) were migrants, who then didn’t have an access to these subsidised apartments…Brunnenviertel was a rough area before. But I moved because of the migrants here, and they were our target group.

[…]

Blocking the (original) redevelopment plan was only possible, because we could get signatures from the market vendors and customers. The vendors participated, because, with high-rises on the market, they would have lost their customers. Most of them wanted to stay and continue to work here. They only had a chance (to keep their businesses), if they acted together…We became sort of a representative office for the vendors that participated and observed the earlier planning process.

While much has been said about the emergence of new actors at different stages in the overall gentrification process, the difference that the newcomers make not only lies on the ‘gentrification types’ (Lees, 2012), but also on their own motivation and expectation, leading to a positive reaction to existing diversity at the local level. Like the early pioneers in the mid-1990s, the grassroots initiatives in Brunnenviertel, which have emerged at the later stages of its transformation, continue to provide opportunities for such encounters to bridge the gap in-between. Emma, an organiser of a cultural initiative, explained what she believes to be the essential role of participatory events that bring people together to build a sense of community:

Outside, we have an exhibition of the art works that the shop owners in the market created themselves. We also regularly organise programmes, where we encourage migrants and also Austrians to do activities together...A lot of people who work here otherwise don’t have opportunities to do things together with Austrians…

Despite some practical limitations, such as a lack of interest among some ‘native’ business owners, these curated contact-zones offer the possibilities of focused interactions between the co-occupants, which provide them a space to express their sense of belonging to the community and a feeling of togetherness in the neighbourhood. Vasilios, a migrant vendor on the market, described the way he utilises these institutionalised spaces:

I’ve been here already for many years, so I know (activists from cultural initiatives) well. We know what they do, and we also participated in some of their programmes. When there are special events, we make food for them too, and after they’re done with work, we sometimes get together…I think it’s good there are programmes that try to bring Austrians and migrants together.

While public intervention shapes the overall conditions of fleeting encounters and experiences with ‘others’ in shared public spaces, the sense of togetherness in these contact-zones is accompanied by the ability of different social groups to sustain their own locations of identity. A Turkish youth, Tolga, who was born in the neighbourhood, said:

Before Yppenplatz was pretty dangerous for (children)…It was really different a couple years ago. There was this one time, when a drunk man tried to do weird stuff to me. Not just him, but there used to be drunk people everywhere here…Now it’s good. There are these new places on Yppenplatz, where you see all sorts of different people. I don’t go to those places over there, but people there are still better than these drunk men who were here before…It doesn’t matter who you are, as long as you don’t make problems with us.

Despite the criticism against the entrepreneurial utilisation of diversity in place-making for exacerbating inequalities (see Peck, 2005), its localised outcome largely depends on the particular institutional capacity and its social context, which are able to preserve existing boundaries of multiple social identities. In fact, what appears to be a converging urban trend among cities may not be identical to the place-specific conditions of living-together that are found at a ‘micro-scale of the neighbourhood’ (Warren and Jones, 2018).

While the institutional context of place-making creates the physical and social conditions, in which specific encounters and experiences with ‘others’ are curated, these do not naturally bring out meaningful interactions between different social groups in shared public spaces. Despite the sense of togetherness and belonging shaped around the new local identity of Brunnenviertel, in fact, more closely-knit interactions continue in places of closed networks in the private realm. Although the renewed urban infrastructure offers strangers shared public spaces to causally mingle without being attentive to one another, the neighbourhood’s semi-public spaces host tighter networks of intimacy, where selective people evaluate and negotiate its physical and social transformation in their own ways.

At the centre of Brunnenviertel’s everyday scene, stands a café, where public meetings were held during the formative years of its renewal process. This famous social club that embodies the neighbourhood’s vibrant multicultural image offers a small-scale meeting ground for young ‘natives’, services for newly arrived migrants and refugees, and platforms for grassroots local initiatives. While the adjacent public square features fleeting encounters between strangers, social interactions in this semi-public space are limited to a specific social group, who project their own perception of diversity in Brunnenviertel. For Ervin, a regular and a local activist of Iranian descent, having meaningful contacts with ‘others’ is not what Brunnenviertel’s local culture is all about.

Why you assume that I would have contact with (other) migrants? I meet different people in Brunnenviertel all the time, but I don’t try to build a relationship with them. I don’t think they want that either. We just simply live in the same area together. Why would we suddenly try to become friends, when we have nothing in common? Here, I’m with friends. But out on the market, for example, I buy what I want, and that’s it!

Nina, a young professional from the same friendship circle, added and marked some areas in the neighbourhood she would purposefully avoid walking into, because these areas represent spaces that belong to a particular group of the residents and visitors.

I don’t think multiculturalism is about people going around making friends with different people. For example, I only come to this café, because I meet friends and other like-minded people. I think what’s more important is that we all live here, and we share the neighbourhood together.

[…]

I’m personally happy that I don’t interact with different people in Brunnenviertel. I’m fine with them, but they’re just some people who I would just not…Like some of the places you mentioned down the market. Only old boring ‘natives’ go there. Who knows? Maybe they are racist (or) right-wing.

Social distance between different social groups here emerge from divergent geographies of emotion, and exclusiveness of small-scale local places, although this lies in tandem with a broader feeling of togetherness that they occupy a shared urban infrastructure. While they recognise diversity as an intrinsic element to the urban fabric that constitutes the unique local culture, the occupants of semi-public spaces actively negotiate the institutional vision of multicultural Brunnenviertel on their own terms. Sociability that the young regulars perform in this particular place emerges from their active negotiation with the local grammar of diversity, which enables them to territorialise their own space of intimacy in close proximity to ‘others’.

At the mid-section of the street market, old ‘natives’ stand around a small kiosk from early morning to the market closing time in the late evening. Most customers at one of the last two remaining ‘native’ establishments on the market seldom venture into other parts of the neighbourhood, as they perceive this location to be the only place, where they can afford to have drinks and spend time with their friends. Frank, an old regular, considers this as ‘his’ place, where he can ‘meet old friends’, while ‘there are no places for (him) at this hour’ in the neighbourhood.

I’m too old for those – how do you say, hip?... – places up there. I could get some beers, then go sit on the square, but it’s too late now…Otherwise, (bars on Yppenplatz) are too expensive for me. Also, it’s more your kind of place, isn’t it? I don’t mind it. It’s nice to have young people like you here. But, look at me! I’m too old for that.

As Frank’s perception of certain locations in Brunnenviertel points out, his recognition of togetherness follows that of plurality that he cohabits in a shared urban environment. He, who recognises the neighbourhood as much plural as communal, built his social life around the site of his local attachment in negotiation with the political language of diversity that accompanies the image of an artsy, cultural, and consumption urban space. For Anna, a waitress at another ‘native’ café, young ‘natives’, whom she describes as ‘artists’, are nothing more than fellow ‘neighbours’, although she has no personal contact with them, nor she ever visits places that she deems too artsy for her:

Have I ever been to the places on Yppenplatz? No. Aren’t they supposed to be only for artists? They never come here, but I wouldn’t mind them…We’re like neighbours, who don’t talk to each other…Although during the day a lot of (migrants) who work on the market come. They come in, have a cup of tea, then go to work again.

While different groups understand the pluralistic composition within the neighbourhood’s shared public spaces that they co-occupy with others, their capacity to build their separated, yet interconnected, social worlds around the politics of place-making has engendered a ‘dispersed sense of the plural communal’ (Amin, 2012), whose ‘difference’ is recognised and its boundaries are respected. For Hakan and Cengiz, two employees at a Turkish butcher shop, living together in the distance is a practical decision that resonates with the image of a vibrant multicultural street market. Hassan, who spoke better German, said:

We only work here. After work, we go back to where we live. So, no. We usually don’t spend so much time here. Only during the break. I can’t say a lot about Yppenplatz, but Brunnenviertel is good, because business is good…(Our customers) are mostly Arabs and Turks, but we also have a few Austrian customers. (Arabic customers) sometimes make problems. But it’s okay. We make a lot of money…

In Brunnenviertel, the bottom-linked design of its urban renewal, connecting the diverse competences of public institutions, grassroots initiatives and residents, garnered local knowledge and experience for a cohesive local multiculture, in which the place-specific needs of existing diversity are accommodated, and the problems of existing inequalities are addressed. One particular enabling context behind this was the overarching structure of stakeholder engagement that enabled the local Urban Renewal Office to supervise diverse thematic renewal programmes and coordinate interactions between grassroots- and institutional actors, which were previously beyond their institutional capacity. The active role of the district authorities in financing the renewal plan allowed the local Urban Renewal Office to formulate a comprehensive renewal plan that exceeded their formal responsibility (e.g. housing renovation and traffic regulation). Furthermore, this organizational realignment assigned the local Urban Renewal Office to a steering role, connecting informal grassroots activities with various institutional actors (e.g. the Municipal Departments, political representatives, and economic promotion agencies) in both the planning and decision-making process.

At the micro-level, this was sustained by the local capacity to host multiple social identities of different social groups without imposing a singular narrative of diversity. The spatial availability of multiple social identities is instrumental in place-making of a diverse neighbourhood, because the preservation of places and spaces, where these identities can be projected, averts assimilation of the local grammar of pluralism into the elitist celebration of ‘creative’ difference (Pemberton and Phillimore, 2018). Indeed, social distance between the co-occupants of Brunnenviertel is an indispensable feature of the daily life that sustains the mundane nature of everyday encounters and experiences with ‘others’. The ambiguity between togetherness and distance, differs from ‘selective belonging’ (Watt, 2009) or ‘social tectonics’ (Butler and Robson, 2001) in gentrifying neighbourhoods studied elsewhere, where different social groups disassociate themselves from one another to construct their social identities. In contrast, the presence of ‘others’ – whether ‘new’ or ‘old’, or ‘native’ or ‘foreign’, constitutes the central node between the different spatial orders in Brunnenviertel that allow people to maintain their elective social circles, of which ‘difference’ they are aware of, and of which boundaries they respect enough not to trespass.

## Conclusion

The ordinariness of living-with-difference, yet in the distance, characterises the everyday scene of the Vienna’s ‘multicultural heart’, where a diverse range of stakeholders across multiple scales built a communal local culture over an extended period of time within the wider institutional context of multilevel place-making. The institutional space, in which divergent knowledge and competences of institutions and actors were configured and their instruments were implemented, produced the specific everyday conditions of living-with-difference, where their diverse interests are accommodated on equal terms without breaching their respective boundaries. In turn, the local ability to form intimate social lives around the politics of place-making sustains its habitual – not, imposed – coexistence, whose ‘difference’ is a trivial feature of its urban fabric. The bottom-linked creation of new contact spaces in a multiethnic neighbourhood that had long been regarded as a segregated problem zone provides a site of positive encounters and experiences with neighbourhood change, around which diverse groups and individuals forge a new sense of togetherness and belonging. From a policy perspective, this is a matter of practical importance, as it hinders exclusive claims of ownership by privileged stakeholders that could reinforce already existing inequalities. From a local perspective, this is a matter of individual and collective well-being, as it gives them a sense of pride in the spaces of everyday life that undermines contentious dividedness in their changing social surroundings.

Before the ‘diversity turn’ in the literature, the shifting dynamics of everyday life in globalising cities have long captivated scholars across different academic disciplines. A review of different streams of work is therefore necessary, as a vast amount of literature has so far brought to intense discussions with conflicting, yet sometimes overlapping, arguments on how strangers in the city negotiate their changing social and physical environment in the everyday scene. While these key streams of debate emerge from different academic traditions and suggest different hypotheses on why, where, and how we socially interact with ‘difference’, these equally stress the need for a place-based approach to the situated contexts of diversity in everyday settings. However, there is a limit in the extant research, as to which institutional principles, instruments and mechanisms can actually contribute to place-based processes and outcomes of place-making within a specific contextual setting. So far, how this contextual embeddedness affects the everyday way, in which strangers form their social world and forge their relations with ‘others’ remain understudied. While the demographic and spatial compositions of the city’s diverse place constitute an important element of living-with-difference, these alone provide insufficient information about the complex reality of the multilevel governance process behind the politics of place-making.

As the example of Soft Urban Renewal in Brunnenviertel shows, the specific design context of formulating and implementing the multilevel place-making process bears significance for the local durability and sustainability of the local culture. In contrast, incongruent mixes of existing institutions and actors with divergent knowledge, competences and interests may reinforce negative spillovers of neighbourhood regeneration, creating social tensions and conflicts between old and new residents. Without such rules and regulations guiding living-with-difference, they may find themselves in competing positions within the social hierarchy of a divided urban space, as mere co-presence in ‘neglected public spaces with rudimentary or exclusionary rules of regulation breed(s) social pathologies of anxiety and avoidance’ (Amin, 2012). This was, and still is, true for cities that lack a strong regulatory framework to mitigate market-related factors affecting the process and outcome of urban renewal, from which negative encounters and experiences with neighbourhood change emerge (Clark and Wise, 2018).

Indeed, the place-specific mode of living-with-difference in Brunnenviertel show the local ability and availability to form positive encounters and experiences that emerges from the city’s specific regulatory capacity, creating complementarity between intervention from top-down and participation from bottom-up in the multilevel renewal process. On the one hand, strong public intervention mattered for providing political and financial resources to preserve existing places of multiple social identities, where their closed interactions with own social groups could be maintained. On the other hand, the active engagement of grassroots initiatives in the place-making process, enabled by significant support from top-down, created a new local image based on actually existing diversity of the neighbourhood, around which the residents formed a new sense of belonging. It is this key role played by a bottom-linked policy design of neighbourhood regeneration, which guide different social groups to perceive ‘difference’ in a prosaic fashion, yet able to respect their boundaries in a broader realm of public life. Here, a sense of belonging and sharedness operate in tandem with social distance, which grants different social groups multiple local ownerships within a shared urban infrastructure, where habitual coexistence between differences do not breach the respective territories of multiple identities.
